# Assessing the incremental costs and savings of introducing electronic immunization registries and stock management systems: evidence from the better immunization data initiative in Tanzania and Zambia

**DOI:** 10.11604/pamj.supp.2020.35.1.17804

**Published:** 2020-02-12

**Authors:** Mercy Mvundura, Laura Di Giorgio, Elisabeth Vodicka, Robert Kindoli, Chipo Zulu

**Affiliations:** 1 Medical Devices and Health Technologies Program, PATH, Seattle, USA; 2 Center for Vaccine Innovation and Access, PATH, Seattle, USA; 3 Country Program Office, PATH, Dar Es Salaam, Tanzania; 4 Country Program Office, PATH, Lusaka, Zambia

**Keywords:** Costing, economic evaluation, immunization registry, Expanded Program on Immunization, data, Tanzania, Zambia

## Abstract

**Introduction:**

Poor data quality and use have been identified as key challenges that negatively impact immunization programs in low- and middle-income countries (LMICs). In addition, many LMICs have a shortage of health personnel, and staff available have demanding workloads across several health programs. In order to address these challenges, the Better Immunization Data (BID) Initiative introduced a comprehensive suite of interventions, including an electronic immunization registry aimed at improving the quality, reliability, and use of immunization data in Arusha Region, Tanzania, and Southern Province of Zambia. The objective of this study was to assess the incremental costs of implementing the BID interventions in immunization programs in these two countries.

**Methods:**

We conducted a micro-costing study to estimate the economic costs of service delivery and logistics for the immunization programs with and without the BID interventions in a sample of health facilities and district program offices in each country. Structured questionnaires were used to interview immunization program staff at baseline and post-intervention to assess annual resource utilization and costs. Cost outcomes were reported as annual cost per facility, cost per district and changes in resource costs due to the BID interventions (i.e., costs associated with health worker time, start-up costs, etc.). Sub-group analyses were conducted by health facility to assess variation in costs by volume served and location (rural versus urban). One-way sensitivity analyses were conducted to identify influential parameters. Costs were reported in 2017 US dollars.

**Results:**

In Tanzania, the average annual reduction in resource costs was estimated at US$10,236 (95% confidence interval: $7,606-$14,123) per health facility, while the average annual reduction in resource costs per district was estimated at $6,542. In Zambia, reductions in resource costs were modest at an estimated annual average of $628 (95% confidence interval: $209-$1,467) per health facility and $236 per district. Resource cost reductions were mainly attributable to reductions in time required for immunization service delivery and reporting. One-way sensitivity analyses identified key cost drivers, all related to reductions in health worker time.

**Conclusion:**

The introduction of electronic immunization registries and stock management systems through the BID Initiative was estimated to result in potential time savings in both countries. Health worker time was the area most impacted by the interventions, suggesting that time savings gained could be utilized for patient care. Information generated through this work provides evidence to inform stakeholder decision-making for scale-up of the BID interventions in Tanzania and Zambia and to inform other Low-to-Middle-Income Countries (LMICs) interested in similar interventions.

## Introduction

Immunization has proved to be the most cost-effective public health intervention through reducing childhood mortality and morbidity attributable to vaccine-preventable diseases. Despite immunization being such an effective public health tool, not all children are being reached with the lifesaving vaccines they need [1]. One key challenge faced by immunization programs, especially in the sub-Saharan African region, is the stagnation of coverage rates [2]; coverage rates for the third dose of diphtheria, tetanus and pertussis-containing vaccine have plateaued in the 70% percentile since 2010 [3]. In addition, drop out rates between the first and second dose of measles containing vaccine can be high and this has implications for the ability of countries to achieve disease elimination.

Several factors have been identified as inhibiting immunization program performance improvement, including the poor quality of data and the poor use of existing data [2, 4–7]. Data quality challenges include inconsistencies and inaccuracies in reported data, which impact key program metrics such as target populations and coverage rates. Poor use of data includes failure to use existing data to inform planning, which can result in low product stock or stockouts and delays in transmission of data to program managers. In addition, programs have challenges tracking which children have received which vaccines and hence during campaigns, vaccines are given to all children in the target age group because there is no data to inform the program about which children are fully vaccinated through routine immunization. Embedded in these data challenges are data formats that make it difficult for health workers to easily identify and track children who are due for vaccinations or track children who move from one area to another, which hinders the provision of optimal services to intended recipients. In addition, low- and middle-income countries are plagued by a shortage of health care workers, who lack the infrastructure to effectively and efficiently manage their programs [8].

Given these challenges with immunization program data, there is a global effort to strengthen country immunization systems by supporting the collection of better-quality data and better use of these data to inform program decision-making. One such effort is through the Better Immunization Data (BID) Initiative [9], led by the Ministry of Health, Community Development, Gender, Elderly and Children in Tanzania and the Ministry of Health in Zambia, in partnership with PATH and funded by the Bill & Melinda Gates Foundation. The initiative is designed to shed light on the challenges surrounding data collection, quality, and use and has identified solutions to improving immunization program data - and potentially applying them to other health areas. The BID initiative worked with the governments of Tanzania and Zambia to develop data quality and use solutions, which include a package of interventions that contains an electronic immunization registry with supply chain information, which enables automatic report generation; data use campaigns; online peer support networks and targeted supportive supervision for health workers. These interventions were implemented at the health facility and district levels. Several research studies were conducted to evaluate the impact of the BID initiative, including monitoring and evaluation of the impact of the BID interventions and costing studies. This article focuses on the findings from the costing studies.

Very few studies have evaluated the costs of interventions aimed at improving data quality and use in other countries that have implemented similar interventions. Hence, we sought to provide some evidence on these costs using data from Tanzania and Zambia. Our objective was to estimate the economic costs of immunization program logistics and service delivery before and after the implementation of the BID interventions, and use these data to estimate the incremental costs or savings attributable to the interventions. The findings from this study are intended to inform the scale-up of such interventions within the two countries and across other countries in the region.

## Methods

### Overview of the baseline system and the BID initiative interventions implemented

Table 1 provides an overview of the immunization registry before and after implementation of the BID interventions. At baseline, health facilities in Arusha Region, Tanzania, and Southern Province, Zambia, were using paper immunization registers, tally sheets and vaccine stock ledgers. Child health cards were used to document vaccines given and these cards were kept by caregivers. Monthly immunization reports were compiled manually using paper report templates. Through BID, tablets were provided to health facilities, which contain software for an electronic immunization registry that include functionality for immunization registration, tallying, stock management and reporting. Tablets were provided to all health facilities in Southern Province. Initially, the tablets were provided only to high-volume facilities in Arusha Region, while low-volume facilities implemented a simplified paper system that helped to streamline data entry and reporting. However, by the end of the project, low-volume facilities had adopted the electronic system, due to challenges of the simplified paper version. The electronic registry is integrated with data use interventions, including an online peer network platform (WhatsApp) and provision of data use job aids to health workers. District staff also provided targeted supportive supervision for health workers. A barcode/quick response code was added to child health cards so that health workers can scan the barcode to retrieve the vaccination record for any given child from the registry. The electronic registration system also automatically generates the monthly reports on the standard immunization reporting metrics.

**Table 1:** Sample size for the health facility analysis and overview of the immunization registry system before and with the BID interventions

	Arusha Region, Tanzania		Southern Province, Zambia	
	Pre-intervention	Post-intervention	Pre-intervention	Post-intervention
	n	n	n	n
**Health facilities**				
Total number of health facilities in the region/province	253	253	253	253
Health facilities sampled for costing study^a^	43	34	52	46
**Overview of the immunization registry system**				
Immunization registration	Relied on paper immunization registry	Electronic registration using a tablet	Relied on paper immunization registry	Electronic registration using a tablet
Immunization stock management	Paper-based immunization stock management system	Electronic management of vaccine stock using tablets and bar codes	Paper-based immunization stock management system	Electronic management of vaccine stock using tablets and quick response codes
Reporting	Paper reports submitted in person at the district	Reports automatically generated and submitted electronically	Paper reports submitted in person at the district	Reports automatically generated and submitted electronically

a Fewer facilities were available to participate in interviews at post-intervention compared to baseline. In Tanzania, at the time of the post-intervention assessment some of the facilities were still using the paper system only and so there was no difference from baseline; hence, we did not collect post-intervention data at these facilities. In Zambia, some facilities had not started using the electronic system at the time of post-intervention data collection, so they were also excluded from the post-intervention data collection. The number of facilities sampled at each time point are reported here

### Facility- and district-level costing

We conducted a micro-costing study [10] to estimate the annual economic costs of resources used for immunization logistics and service delivery before and after implementation of the BID initiative in Arusha Region in Tanzania and Southern Province in Zambia. The study focused on the health facilities and districts in which the BID interventions were implemented and hence did not include regions/provinces or the national level.

We developed primary data collection tools to identify resources used for transporting and storing vaccines, staff time for logistics and service delivery, office equipment and communications, and printing and office supplies. Similar questionnaires were used to collect data on the resources used at district level, focusing on activities related to the logistics and management of health facilities. The tools used in the two countries were similar, but adaptations were made to reflect country-specific characteristics of each immunization system.

We collected data from a sample of health facilities in each district and a sample of districts in each region/province. We included 4 of the 7 districts in Arusha Region and 6 of the 13 districts in Southern Province. Health facility sample sizes are shown in Table 1. Baseline and post-intervention data were collected from the same sample of facilities and districts, which we selected using a purposive sampling approach based on key characteristics expected to affect the costs of providing immunization services. These parameters included average number of monthly immunizations dichotomized into low (< 50 children) and high volume (≥ 50 children), location (rural versus urban) and distance from the district immunization office. BID staff administered the questionnaires through in-person interviews at each facility.

At the time of post-intervention data collection, the facilities in Tanzania had been using the electronic immunization registry for an average of 8 months (range 4 to 11 months); in Zambia, the average was 3.5 months (range 1 to 8 months). In addition, at the time of post-intervention data collection, health facilities in both countries were still using the paper-based system as back-up because policy decisions had not yet been made to eliminate the paper system and solely rely on the electronic system. Therefore, we asked health workers to assess the change in resource use under a scenario in which only the electronic system was in use.

### Types of costs included in the costing study

We collected immunization service delivery costs across five main categories: (1) human resources; (2) cold chain equipment; (3) communications, printing and office supplies; (4) facility office equipment and (5) transport. Human resources costs included salaries and per diems for staff working in the immunization program. Staff were asked to self-report the time spent on providing fixed and outreach immunization services, logistics and stock management for the immunization program and data reporting. The costs of cold chain equipment captured the capital costs of refrigerators, freezers, cold boxes, and vaccine carriers used in the immunization program, and the annual costs of electricity or gas to run the cold chain equipment, as relevant. Costs of office equipment and communications included capital costs for computers, tablets, printers, scanners, and other equipment used by the immunization program and communication and printing costs. Finally, transport costs reflected the costs to collect vaccines and immunization supplies from the district and transport them to facilities or to conduct outreach services (hired vehicles, public transportation and capital and fuel costs for vehicles owned and maintained).

### Data analysis

For resources shared with other programs, costs were allocated to the immunization program based on the reported percentage spent or use of the immunization program. Capital costs were annualized using different lifespans: 3 years for office equipment; 5 years for vehicles; and 10 years for cold chain equipment. All local cost data were collected in local currencies and converted into 2017 US dollars using average exchange rates for the year [11]. As necessary, we updated prices for inflation using consumer price indices from the World Bank [12]. Unit prices (Table 2) were obtained from various sources, including local data sources, World Health Organization (WHO) Comprehensive Multi-Year Plans [13], online databases [14–17] and BID Initiative project records.

**Table 2:** Selected unit prices (in 2017 US dollars)

Resource	Tanzania	Zambia
Nurse monthly salary	$549	$532
Nurse assistant monthly salary	$426	$486
District immunization officer monthly salary	$1,098	$773
Truck/Pickup	$70,000	$70,000
Refrigerator (average of brands used)	$2,315	$1,323
Tablet (at district level)	$325	$325
Tablet (at health facility level)	$152	$152
Barcode/Quick response code scanner	$175	$175
Printer (at district level)	$600	$600
Scanner (at district level)	$550	$550
Immunization register	$1.24	$0.50
Stock register	$0.91	$0.50
Fuel costs	$0.84	$1.25
Electricity price per kWh	$0.09	$0.03

kWh: kilowatt hour

Resource use data were combined with unit costs to calculate economic costs at the district and facility levels. All cost estimates (baseline, post-intervention and incremental) were reported as annual economic costs per facility or district. Our incremental cost estimates relied on the cross-sectional data from the two surveys conducted, one at baseline and one at post-intervention. These surveys were conducted at different time points. However, given that time use was self-reported and subject to recall bias, at post-intervention, along with asking survey respondents to estimate time spent on immunization activities with the BID interventions, we asked them to recall and report how much time they had been spending on these same activities before the implementation of the BID interventions. We used these data to provide an alternative set of estimates for the analysis. In addition, we conducted univariate sensitivity analyses to identify the cost drivers and time use for the interventions.

## Results

### Health facility costs

Table 3 shows the economic cost estimates for the health facilities, comparing the costs of the resources used using the data from the baseline and post-intervention surveys. In Tanzania, we estimated at baseline that the annual average economic costs per health facility totaled US$17,318 (95% confidence interval [CI]: $12, 113–$24, 289]. Post-intervention average facility costs were $7,082 [95% CI: $4, 506–$10, 116], reflecting estimated annual savings of $10,235 per facility each year due to efficiencies generated in the immunization supply chain, service delivery, and time spent on immunization activities at the facility level (Table 3). These savings are attributable to reductions in several areas, including human resources costs because of reductions in time spent on immunization reporting and management activities and emergency trips for vaccine resupply, and elimination of printing costs for paper registers and tally sheets not required for the BID interventions. The one cost category that increased because of the BID interventions was office equipment, because of the provision of tablets and barcode or quick response code readers at each health facility. Capital costs for cold chain equipment remained unchanged from pre- to post-intervention because the interventions had no impact on these costs. Using the time use data for baseline and post-intervention based on responses from only the post-intervention survey, we found that for the health facilities in Arusha Region, Tanzania, the estimated savings in salary and per diem costs were $6,642, lower compared to the $10,245 reported when using the responses from the baseline and post-intervention surveys.

**Table 3:** Average annual cost per facility for the immunization program at baseline and post-intervention (in 2017 US dollars)

Parameters	Facilities in Arusha Region, Tanzania			Facilities in Southern Province, Zambia		
	Baseline	Post	Incremental	Baseline	Post	Incremental
	mean (95% CI)	mean (95% CI)	mean (95% CI)	mean (95% CI)	mean (95% CI)	mean (95% CI)
Salaries and per diems	16,468 (11,509, 23,175)	6,223 (3,806, 9,127)	−10,245 (−14,048, −7,703)	4,391 (854, 5,974)	3,663 (2,211, 5,693)	−728 (−291, 1,357)
Cold chain equipment	399 (395, 403)	399 (395, 403)	0 (0, 0)	313 (255, 371)	313 (255, 371)	0 (0, 0)
Transportation	302 (145, 476)	258 (118, 420)	−44 (−28, −56)	534 (341, 739)	520 (337, 715)	−14 (−24, −5)
Office equipment	0 (0, 0)	138 (138, 138)	138 (138, 138)	11 (0, 22)	149 (138, 161)	138 (138, 138)
Printing, Internet, and telephone	148 (63, 235)	63 (49, 78)	−85 (−14, 157)	74 (55, 93)	50 (40, 60)	−24 (−15, −33)
Total per facility	17,318 (12,113, 24,289)	7,082 (4,506, 10,166)	−10,236 (−14,123, −7,606)	5,324 (1,506, 7,209)	4,695 (2,981, 6,999)	−628 (−209, 1,476)

CI: confidence interval

In Zambia, we estimated similar trends as for Tanzania but Zambia’s baseline costs were lower at $5,324 [95% CI: $1,506-$7,209]. Post-intervention costs were estimated at $4,695 for Zambia [95% CI: $2,982-$6,999]; therefore, savings attributable to the BID interventions were smaller (we estimated a savings of approximately $628 per facility each year). This represents a 12% reduction in costs per facility with BID compared to baseline. Similar to Tanzania, the largest savings would be achieved through reduction in staff time on immunization activities. We also estimated a reduction in annual transport costs of $14 per health facility, resulting from a reduction in emergency trips to the district vaccine store to collect vaccines and transportation for immunization-related outreach activities.

**Figure 1:**
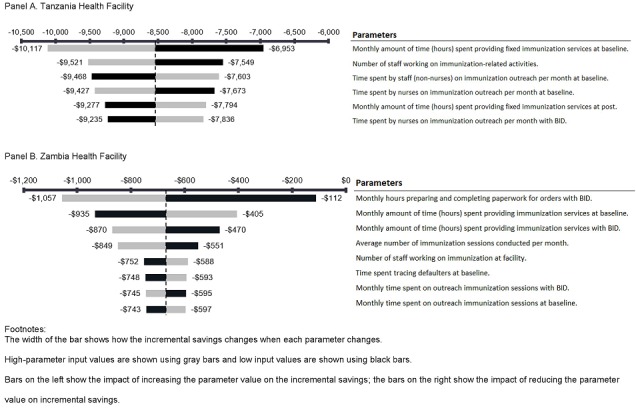
Univariate sensitivity analysis evaluating influential parameters on incremental savings from introducing electronic immunization registries and stock management systems into Tanzania and Zambia

We conducted univariate sensitivity analyses to assess uncertainty and identify influential parameters on changes in resource utilization due to the interventions (Figure 1). Influential parameters were considered any variables with uncertainty ranges wider than 20% of the total incremental change in health facility costs. Across all Tanzanian facilities, time spent on provision of routine and outreach immunization services and total number of staff allocated to the immunization program (stratified by nurses and other staff) appeared to be the largest drivers of the incremental cost per health facility (Figure 1 A). Sensitivity analyses in Zambia derived similar results (Figure 1 B). Incremental costs appeared to be driven by the time spent on paperwork and providing fixed immunization services, as well as the estimated number of immunization sessions per month.

When results were stratified by health facility characteristics -rural versus urban and low volume versus high volume- we found that in Tanzania, there was a smaller variation across health facility categories based on location (results not shown in tables). For example, rural facilities were estimated to save an average of $8,144 [95% CI: $6,812-$9,820] with the introduction of the interventions, compared to $9,423 [95% CI: $5,787-$13,972] savings in urban facilities. The variation was slightly higher based on volume served; we found that low-volume settings were estimated to save approximately $7,683 [95% CI: $5,116-$10,964] per facility compared to $9,367 [95% CI: $7,671-$11,560] in high-volume facilities. In Zambia, the immunization volume categorized as low or high volume resulted in much larger variation between strata, with savings of $275 [95% CI: $205-$298] in low-volume facilities compared to $2,177 [95% CI: $1,408-$3,037] in high-volume facilities. Stratified cost savings were $380 [95% CI: $612 cost savings to $247 increased costs] and $776 [95% CI: $2,218 cost savings to $89 increased costs] in rural and urban facilities, representing 9% and 13% decreases in costs, respectively.

### District-level costs

Table 4 shows the results of the baseline and post-intervention costing analysis at the district level. At baseline, the average logistics and service delivery costs in Tanzania were $23,001 per year, excluding the value of vaccines. Human resources accounted for the largest share of costs, at more than 50%. Based on the responses provided by staff at the district level about the impact of the interventions, we found that the interventions did not have any impact on the costs of cold chain or transport. The BID interventions impacted the costs of the following items: communications, printing and office supplies, office equipment and human resources. The interventions resulted in an average increase in communication costs of $167 per district. Equipment costs increased by about $491 per district because a tablet, printer and scanner were provided to each district immunization office. Other equipment costs were not expected to change with the introduction of the interventions. Human resources costs were the most impacted by the interventions, as district office staff reported a significant reduction in time spent on estimating vaccine needs, processing orders and distributing vaccines to health facilities. A few human resources activities saw an increase in time use, such as supervision and support, due to more time being spent with facilities to provide technical support with the electronic registry. On average, a district office in Tanzania estimated that human resources time valued at $7,200 would be saved per year when compared to the system without the interventions. Overall, we estimated that the rollout of the interventions would result in net savings in the amount of $6,542 per year at the average district office in Arusha Region, Tanzania. This represents a 28% reduction in costs after the introduction of the interventions.

**Table 4:** Average annual cost per district for the immunization program at baseline and post-intervention (in 2017 US dollars)

Parameters	Districts in Arusha Region, Tanzania			Districts in Southern Province, Zambia		
	Baseline	Post	Incremental	Baseline	Post	Incremental
	mean (SD)	mean (SD)	mean (SD)	mean (SD)	mean (SD)	mean (SD)
Salaries and per diems	$13,655 ($7,121, $21,061)	$6,456 ($2,452, $10,362)	−$7,200 (−$10,699, −$2,807)	$3,693 ($1,082, $6,23)	$2,904 ($1,043, $6,559)	$−789 (−$2,116, $276)
Cold chain equipment	$2,258 ($1,046, $3,847)	$2,258 ($1,046, $3,847)	$0 ($0)	$855 ($470, $1,364)	$855 ($470, $1,364)	$0 ($0)
Printing, Internet, and telephone	$269 ($110, $503)	$436 ($298, $688)	$167 ($118, $222)	$1,188 ($59, $2,416)	$1,250 ($200, $2,365)	$62 (−$51, $141)
Office equipment	$68 ($63, $70)	$559 ($554, $561)	$491 ($491, $491)	$19 ($7, $39)	$510 ($498, $530)	$491 ($491, $491)
Transport costs	$6,751 ($2,499, $11,607)	$6,751 ($2,599, $11,607)	$0 ($0, $0)	$11,823 ($2,524, $16,609)	$$11,823 ($2,524, $16,609)	$0 ($0)
Total per district	$23,001 ($11,064, $35,718)	$16,459 ($8,891, $25,695)	−$6,542 (−$10,023, −$2,173)	$17,578 ($6,595, $23,724)	$17,341 ($7,280, $22,178)	−$236 ($1,456, $800)

The estimated savings in Zambia from the implementation of the interventions were more modest. The new equipment increased district annual costs by $491 and communication costs by $62. Most of the savings were derived from reduced labor time and fewer printing costs for immunization registers, stock ledgers and tally sheets. We estimated that time valued at $789 would be saved per year by staff at each district office. Overall, the rollout of the interventions was estimated to result in annual net savings of $236 for the average district office in Southern Province.

## Discussion

This study aimed at estimating the cost implications of introducing electronic immunization registries and stock management systems in Arusha Region, Tanzania, and Southern Province, Zambia. In both countries, we found that electronic systems may result in savings compared to paper immunization and stock registers. Savings were mostly attributable to reduction in health workers’ time spent on immunization activities, such as administrative tasks and reporting. Efficiencies gained due to electronic registration and reporting were, as expected, higher in absolute terms in high-volume facilities compared to low-volume facilities.

To our knowledge, this study is the first to estimate the savings to be realized using electronic immunization registries and stock management systems in resource-limited settings. Few studies published in the literature evaluated interventions similar to those introduced through the BID Initiative [18–24], most of which were based in high-income countries. Due to the vast differences in financial resources, immunization programming, and health care delivery systems, these findings provide little opportunity for comparison or use in decision-making for immunization programs in sub-Saharan Africa. In terms of benefits, the US-based studies identified increases in administrative efficiency such as the reduction in reporting burden. We found similar efficiencies from reductions in time spent on daily registration during immunization sessions and on monthly reporting. The dearth of studies in low- and middle-income countries highlights a lack of evidence on the costs and benefits of electronic immunization registries and relevant immunization interventions in these countries.

While not having a direct financial implication for health ministry budgets, the reduction in health worker time represents an important finding and suggests that human resources could be freed up at health facilities so that staff could spend more time on patient care rather than administrative tasks. Also, given the competing time demands of health workers who work across different programs, the benefits of the time savings could be spread to other programs. At the district level, the time saved from the automatic generation of monthly reports is expected to allow district immunization officers to divert energy to other activities, such as supervision.

We found that the estimated savings were much larger for districts and health facilities in Arusha Region, Tanzania, than those in Southern Province, Zambia. While there could be other reasons for these differences, we suspect that the shorter evaluation time in Zambia (between the deployment of the BID initiative interventions and the collection of the post-intervention costing data) may partially explain these findings. In fact, we hypothesize that staff were still adjusting to the new system in Zambia and hence had not gleaned the full benefits of the new system.

Our study has several limitations. First, the cost estimates are not representative, as a purposive sampling approach was used and samples were relatively small. To address this limitation, we conducted sensitivity analyses and varied input values over low and high ranges to assess how the cost implications would differ under varying assumptions. Second, the time between rollout of the interventions and post-intervention data collection was short, especially in Zambia. This choice was driven by the project timelines and the broader delays experienced during rollout of the interventions. Therefore, estimates reported here are likely to capture learning costs and thus underestimate savings from the BID interventions. A longer-term evaluation of the BID initiative may be warranted to provide more accurate estimates of its cost implications. Third, all time use data included in this analysis were self-reported and thus may have been under- or overestimated. Also, at the time of post-intervention data collection, countries were continuing to use both their paper-based system and the electronic system, making it difficult to assess system changes. This is because the relevant ministries of health had not yet made the decision to solely rely on the electronic system. As a consequence, we relied on staff’s assessment of what their time use would be if they were using only the electronic system. Fourth, due to the lack of data or complexity in assessing them, the study did not include all benefits that could result from the BID interventions, further underestimating its benefits. For example, potential benefits in terms of a decrease in the number of stockouts and wastage, better forecasting, more timely immunizations and higher coverage, and improved decision-making could not be taken into account because of the shortness of time between deployment and evaluation. In addition, our ability to capture the costs of the immunization program relied on the availability and quality of the data. Finally, in this study we did not include the upfront costs of implementing the BID interventions, such as the system development costs or the costs of rolling out the BID interventions to facilities and districts and the costs of maintaining the system. These costs will be reported in a separate analysis.

## Conclusion

The introduction of electronic immunization registries and stock management systems through the BID initiative was estimated to be cost saving in Tanzania and Zambia. These savings were primarily due to time efficiencies and associated staff cost savings. Information generated through this work provides evidence for key stakeholders in Tanzania and Zambia to inform decision-making for the scale-up of the BID interventions in these countries and to inform decisions in other countries that may be interested in similar interventions.

### What is known about this topic

- Poor data quality and low data use are key challenges that negatively impact immunization programs in low- and middle-income countries;
- Electronic immunization registries (EIRs) together with related data use interventions can be a solution for these challenges.

### What this study adds

- Evidence on the incremental costs of implementing EIRs and data use interventions in immunization programs in Tanzania and Zambia;
- The study found that the interventions introduced resulted in savings in health worker time.

## Competing interests

The authors declare no competing interests.
